# Using Visual Methods to Understand Physical Activity Maintenance following Cardiac Rehabilitation

**DOI:** 10.1371/journal.pone.0138218

**Published:** 2015-09-18

**Authors:** Sarah J. Hardcastle, Keira McNamara, Larette Tritton

**Affiliations:** 1 Health Psychology and Behavioural Medicine Research Group, School of Psychology and Speech Pathology, Curtin University, Perth, Australia; 2 Department of Clinical Sciences, Brunel University, London, United Kingdom; 3 School of Sport and Service Management, University of Brighton, Brighton, United Kingdom; University of Stirling, UNITED KINGDOM

## Abstract

Few studies have explored the factors associated with long-term maintenance of exercise following cardiac rehabilitation. The present study used auto-photography and interviews to explore the factors that influence motivation and continued participation in physical activity among post cardiac rehabilitation patients. Twenty-three semi-structured interviews were conducted alongside participant-selected photographs or drawings with participants that had continued participation in physical activity for at least two years following the cardiac rehabilitation programme. Participants were recruited from circuit training classes in East Sussex in the UK. Thematic content analysis revealed seven main themes: *fear of death and ill health avoidance*, *critical incidents*, *overcoming aging*, *social influences*, *being able to enjoy life*, *provision of routine and structure*, *enjoyment and psychological well-being*. Fear of death, illness avoidance, overcoming aging, and being able to enjoy life were powerful motives for continued participation in exercise. The social nature of the exercise class was also identified as a key facilitator of continued participation. Group-based exercise suited those that continued exercise participation post cardiac rehabilitation and fostered adherence.

## Introduction

Cardiac rehabilitation interventions have been shown to improve risk factor management and life expectancy in patients with coronary heart disease [1–3]. However, despite the benefits, the rate of cardiac rehabilitation uptake is low ranging between 15–50% [4–6]. Yohannes et al [7] reported early cardiac rehabilitation drop out with 22% of patients ceasing participation within the first two weeks. Daly et al [8] found that only 33% continue with cardiac rehabilitation programmes after 6 months. Sustaining habitual physical activity participation is necessary following cardiac rehabilitation completion to maintain the associated health benefits. However, findings indicate that many patients do not maintain physical activity participation following programme completion [9–12].

The factors influencing exercise maintenance in the minority of patients that do adhere to exercise following cardiac rehabilitation are unclear. Much of the previous work on cardiac rehabilitation has examined methods to increase referral to programmes [13–15] or the determinants of short-term adherence to programmes [16, 17]. Constructs such as motivation, self-efficacy, outcome expectancy, perceived behavioural control, health status, intention, and past experience of physical activity appear to explain some of the variance in exercise adherence [8, 18, 19].

Clark and colleagues [20] conducted a review and meta-synthesis of studies exploring the factors influencing participation in cardiac rehabilitation following referral and initial attendance and found that with the exception of perceived benefits of exercise, the main facilitators of exercise participation were contextual factors including social networks and benefits. Numerous studies in the review reported that the social and group-based nature of exercise programmes fostered continued participation [21–25]. The social dimensions were valued because they enhanced social camaraderie with other participants [25, 26] and provided mutual social support [27].

The review by Clark and colleagues [20] is the first review identifying the factors that influence patients’ ongoing participation in cardiac rehabilitation programmes after referral and initial access. However, the review does not focus on exercise maintenance following cessation of a formal cardiac rehabilitation programme and older adults were underrepresented in the studies. Consequently, there is a lack of research exploring maintenance of exercise amongst older individuals who adhere for more than 12-months and data on the motives of this population for leading a physically active lifestyle would be valuable to inform the development of future interventions targeting insufficiently active cardiac rehabilitation patients. For the purposes of the present study, we adopted Marcus et al.’s [28] definition of successful maintenance of physical activity change. Successful maintenance refers to situations where previously sedentary individuals, who increased their level of physical activity during a physical activity programme, are still regularly exercising for at least six months after programme cessation.

Very little qualitative research has been conducted to explore physical activity experiences of individuals following participation in cardiac rehabilitation. Two exceptions to this are studies by Fleury and colleagues [29] and Rogerson et al. [30]. Fleury et al. ([29] found intrapersonal barriers such as perceived physical condition, a lack of motivation and interest, and competing demands influenced cessation of physical activity following cardiac rehabilitation. Rogerson et al [30] found that facilitators of exercise following cardiac rehabilitation included having a reason for exercising, being able to identify the psychological benefits of exercise and having positive social support [30].

Qualitative approaches are best suited to understand and explore participant perceptions of health and physical activity, and their decision to maintain regular exercise in the long term. Exploring the perspectives of those that have made a successful transition from cardiac rehabilitation completion to physical activity maintenance could also provide valuable insight as to which theories of behaviour change to adopt as a framework for future interventions. Qualitative approaches that are, by nature, inductive or ‘bottom-up’ rather than deductive or ‘top down’ are useful for unearthing how people change and eliciting the factors that may foster behavioural adherence. Qualitative approaches may be considered a starting point for considering the potential mechanisms of change and applied behaviour change techniques to adopt to facilitate behaviour change [31]. The present study used photo-elicitation (auto-photography) as the main form of data collection. Photo-elicitation is a qualitative research technique that uses a photograph or drawing to stimulate discussion [32]. Auto-photography involves providing a camera to the participant to choose images that answer research driven questions and share with investigators. Auto-photography can provide an additional layer of insight into an individual’s life by enabling researchers to view the world through the participant’s eyes, and in doing so provides participants with a sense of agency and a voice. Furthermore, photographs “embody a way of seeing and can help illuminate what people value, what images they prefer, how they make sense of the world and how they conceive others” (p.168) [33,]. The potential for using visual methods with interviews has been gaining increased attention [34, 35]. Photographs have been used in conjunction with interviews to stimulate enriched storytelling of health-related experiences such as understanding self-management in chronic illness [36] and the experience of homelessness [37]. In the context of physical activity, there are a handful of studies in which researchers have used photo-elicitation as a means to understand the factors influencing physical activity and health behaviour change [33, 38–41]. To our knowledge, none have used auto-photography to explore motives underlying health-behaviour motivation and adherence following cardiac rehabilitation. The aim of the present study was to explore the factors that influence motivation and commitment to continued exercise following participation in a cardiac rehabilitation programme using auto-photography.

## Method

### Participants and Recruitment

Twenty-three participants (five females and eighteen males, *M* age = 72.3 years, SD = 7.3) who had completed the four phases of cardiac rehabilitation were recruited from post phase four circuit based exercise classes at a leisure centre in East Sussex. Participants were recruited on the basis that they had completed a cardiac rehabilitation community programme at least two years previously and were willing to participate. Fifty participants were invited to participate in the study. The main reason for declines to participate was time constraints and finding the auto-photography task rather daunting. The Researchers undertook the auto-photography task themselves in order to provide participants with an example. Although most participants were happy to attempt the task, at least two declined to participate on the basis that they felt the auto-photography task was too intrusive. Participants were recruited informally at the end of the exercise session by the researchers helping as volunteers assist with the exercise sessions.

#### Ethical Statement

Ethical approval was obtained from the University of Brighton Human Research Ethics Committee prior to data collection. Participants signed consent forms to confirm that they were fully informed about the purpose of the study and understood their participation rights (e.g., voluntary participation, right of withdrawal, and confidentiality of the data). It was explained that permission must be granted by any individual appearing in a photograph prior to sharing the image with the researchers and that such images may be reproduced in publications emerging from the research. All participants gave consent for the researchers to use their drawings and photographs in the write-up of the study and subsequent publications. The individuals in this manuscript have given written informed consent (as outlined in PLOS consent form) to publish these case details. Participants were informed that pseudonyms would be used in any reporting of the data to protect their identity. The interview transcripts are available for viewing at http://osf.io/vuzkr/. The researchers that collected the data were not employed by the leisure centre and did not lead the exercise sessions; they were volunteers. The participants were reminded that the researchers were not directly involved with the exercise programme and were asked to be candid and open in deciding which photos or picture to show and in discussing their motives for continued exercise participation.

#### Procedure

Two researchers collected the data between January 2012 and March 2014. Both researchers had spent at least 12-months as volunteers in the setting assisting the exercise leader with the exercise classes. The researchers had developed a rapport with participants during informal conversations prior to the study. The researchers conducted semi-structured interviews in conjunction with participant-created auto-photography. The method of auto-photography involved the participant taking photos (or drawing pictures) that represent who they were in relation to a given phenomenon or topic (for example, ‘what health means to me’) [42]. Arguably, the creative process helps participants to reflect deeply on topics [43] and the use of photographs/pictures or drawings can assist people to organise their personal thoughts more effectively [44]. In the case of the present study, auto-photography was used as a novel approach to further explore motives for exercise maintenance following completion of cardiac rehabilitation. Once participants had agreed to participate and provided consent, they were invited to find pictures, photograph, or draw between three and five photographs/pictures to help explain visually what exercise meant to them. Thirteen and ten participants participated in the auto-photography and drawing tasks respectively. The following instructions were provided to all participants with the visual task:

*When choosing your three-five photographs or drawings for this study*, *I would like you to consider a couple of questions that could influence your choice*. *These questions should help you think about why you exercise and its importance to you*.

*What does exercise mean/or not mean to you*?

*What does being healthy/unhealthy mean to you*?

*Why do you participate in the circuit class*?

*Why are you different to other people who don’t exercise*, *what makes you different*?

**Fig 1:**
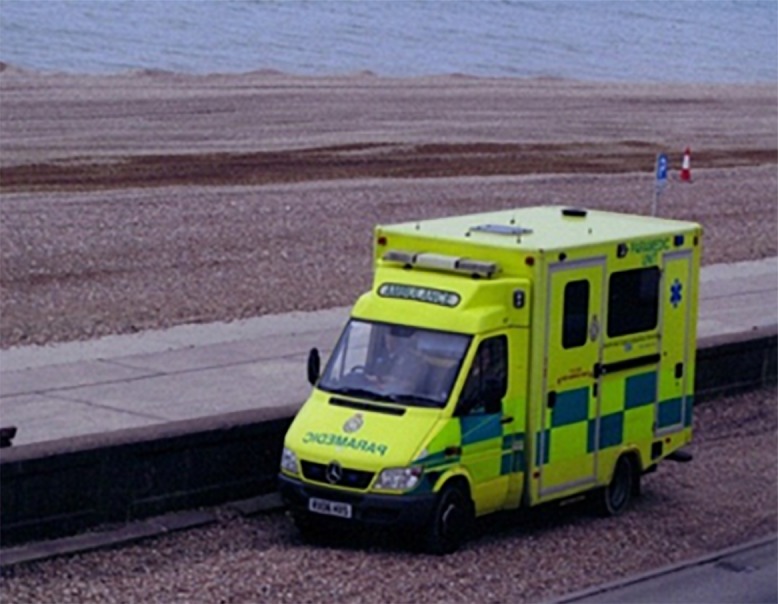
Fear of being in Hospital.

Participants were given a period of two weeks to take, draw or find pictures that related to the questions in Fig 1. After this period, the researchers contacted each participant to arrange a face-to-face interview to discuss the reasons for the selection of photographs/pictures, their meanings and to explore the reasons underlying their continued exercise participation. Data collection ceased at the point when no new information was gained and data saturation was reached [42].

#### Semi-Structured Interviews

Semi-structured one-on-one interviews [42] were conducted with participants to explore the factors influencing their maintenance of physical activity. In this process, the participants’ personal photographs, pictures or drawings were used as a resource to ask questions and explore the personal meanings in the visual data. For example: “Please could you take me through your selected pictures and explain them to me?” These interviews were audiotaped with the consent of the participant and transcribed verbatim. The photographs/ pictures or drawings not only provided a stimulant for generating discussions but provided a foundation that created conversation and prevented the researcher leading discussions towards expectant motives and themes.

### Data Analysis

In the present study visual images were used primarily as an elicitation role in interviews. Therefore, the visual data were analysed alongside the participant’s description, therefore improving the trustworthiness of interpretations since these were not solely based on the researcher’s understandings [45].

Data were analysed by each researcher and the first author using inductive thematic content analysis [46]. Several steps were involved in the process of analysis. The first step involved immersion. During the immersion process the transcripts were read carefully alongside the photographs and drawings several times to identify participants’ meanings and experiences. The second step involved attaching codes to salient text segments. The initial coding was systematically conducted on the entire data set. During this process, several meetings were held to jointly look through each transcript and set of photographs or drawings and agree on coding. The third step involved the identification of themes at a broader level and examining whether codes may be combined to form an overarching theme. During these processes, inductive analysis was used to identify themes that emerge directly from the visual and interview data linked to reasons for continued physical activity participation. This is in contrast to a deductive approach whereby predetermined themes are used to organize quotes. It is recognized, however, that although there is an attempt to be ‘open’ to the data in terms of emerging themes, the subsequent themes developed and interpretations offered will also be influenced by the researchers’ prior knowledge and in relation to previous research and theoretical constructs [46, 47]. The identification of themes involved producing thematic maps to decide which codes had a common thread and could be combined to form an overarching theme. The final step involved reviewing themes, cross-checking for overlap and differences and finally defining and naming themes.

#### Credibility and Quality

We have attempted to demonstrate credibility and quality in the analysis in four ways. First, the researchers have prolonged engagement in the setting prior to the study and such engagement has been demonstrated to increase data credibility [48, 49]. Second, since the auto-photography method adopted was participant centred and created, this helped to minimise researcher led questions and prompts. However, this must be cast in the light of the fact that the researchers determined the number of drawings or photographs to present and the questions that formed the basis for the photos or drawings. On the other hand, it was deemed necessary to provide some structure to the task given to participants and keep them focused. Third, data analysis was undertaken by two researchers who discussed each transcript and set of pictures in turn and agreed on codes and themes across the data set. Fourth, we have used lengthy quotes and ‘evocative representation’ (through pictures) and multivocality in the reporting of themes to allow reader judgement of interpretations.

## Results

The purpose of this study was to explore the factors underlying maintenance of physical activity at two years or more post cardiac rehabilitation. Table 1 provides an overview of the sample by pseudonym, age, types of pictures and associated themes provided by participants. The thematic content analysis of the data identified seven main themes connected to the dimensions underlying exercise maintenance: *fear of death and ill health avoidance*, *critical incidents*, *overcoming aging*, *social influences*, *being able to enjoy life*, *provision of routine and structure*, *enjoyment and improved mental well-being*. Each theme and its sub-themes will be discussed in turn.

**Table 1:** Overview of Sample and types of Pictures and associated themes.

Name (Pseudonym)	Age	Picture Types and associated Themes
Jack	80	Black and White photos of Jack with various sports teams, namely cricket (*being able to*)
David	69	Ambulance (*fear*); hospital (*fear*); the exercise class (*provision of routine and structure*); coffee with other participants (*social influences*)
Simon	70	Family, holidays, acting on stage (*being able to*)
Matthew	71	Hospital (*fear*); family, hiking (*being able to*)
Roger	78	Family and grandchildren (*being able to*); at a bar with a friend (*critical incidents*)
Andrew	83	Ambulance (*fea*r); holidays abroad, family (*being able to*)
Joan	74	Countryside, walking, family (*being able to*)
Barbara	67	Hospital (*fear*); home (*independence*), friends and family *(being able to*)
Peter	87	Wife, letter from the queen for diamond wedding anniversary (*overcoming aging*)
Joyce	73	Walking dogs (*being able to*); printouts of physical activity and healthy heart guidelines from the internet.
Julie	64	Holidays and son’s wedding (*being able to*); Wimbledon final (*critical incidents*)
Ian	77	Family and holidays abroad (*being able to enjoy the good life*)
Karen	82	Holidays abroad, golf, walking (*being able to enjoy the good life*); family (*being able to spend time with family*)
Paul	65	Family, holidays, travel (*being able to*); drawing of a house (*independence*); socialising with other exercisers(*friendship and support*, *enjoyment*)
Carl	69	Weighing scales (*fear/ functional fitness*); pet dog (*being able to*); a circle of individuals holding hands (*friendship and support*); a thumbs up (*functional fitness*)
Mark	82	Pronounced biceps (*functional fitness*), bicycle (*being able to*); pet dog (*being able to*); aeroplane (*being able to travel*); shaking hands (*being in the same boat*)
Jason	71	Tea and biscuits(*friendship and support*); group exercise (*being in the same boat/provision of structure*); heart & kidneys (*fear*); wheelchair and walking (*independence*); relaxing in the sun (*being able to*)
Bill	70	People sitting around a table (*being in the same boat/friendship and support*); grumpy to happy face (*psychological well-being*); participant and granddaughter playing tennis (*being able to*); bicycle (*being able to*)
Harry	65	a heart (*fear*); exercising with others (*structure and routine/enjoyment*; a ‘helping’ hand (*being in the same boat*); a heart shaped face smiling (*psychological well-being*)
William	76	A heart (*fear*); wife, grandchildren (*being able to*); smiley faces (*fun and friendship*); pills and medication (*being in the same boat*)
Robert	67	Two having a coffee together (*friendship and support*); fat stick man and a slim stickman (*lose weight*); sad stickman to happy stickman (*psychological well-being*); ears (*listening to others in the same boat*)
Jim	63	Damaged heart (*fear*); bulging waistline (*lose weight*); mug of coffee, sad and smiley faces (*structure and routine/ friendship and support*)
Patrick	59	Sad and happy faces, the sun (*enjoyment and psychological well-being*); defibrillator (*fear*), family (*becoming a burden*)

Note: The associated themes appear in the brackets.

### Fear of death and ill-health avoidance

One of the key themes that emerged was fear of death and avoidance of ill-health. This was depicted in the photographs that participants provided (See Fig 1 provided by Matthew). Andrew (Aged, 83) split his photographs into two groups and explained: “I want less of that (the ambulance outside his house two days previously) and more of all the other photographs…95% of the motivation is to avoid any other health problems”. David (Aged, 69) and Barbara (Aged, 67) also took photographs of an ambulance and a hospital respectively to demonstrate their main motive for continued exercise participation. Likewise, Patrick (Aged, 59) pointed to his sketch of a person and a defibrillator:

*that’s me or that’s a human being and this is a heart attack and these are paddles*, *jump leads life starters erm and fundamentally in the final analysis*, *exercise is about that… it’s also about preserving life and avoiding relapse into further heart episodes avoiding death*...*fundamentally that is all about avoiding this avoiding further relapse further illness further heart deterioration*

Several participants drew pictures of damaged hearts (Harry, William, Jason and Jim). Ill-health avoidance and health concerns were among the forces driving motivation for most participants. Jason placed importance on exercising to maintain his health. He stated *“why I come to the exercise classes is to keep healthy… I know if the heart packs up that’s it*, *I need to keep it going*” (Jason, aged 71).

### Critical incidents

Critical incidents were portrayed in different forms particularly focusing on the loss of loved ones. A photograph that Roger brought in was of himself and a friend having a drink in a bar on holiday. However, despite the seemingly happy picture, it depicted sadness and death. Roger describes “*A good friend*, *but he died just recently…made me see that he didn’t look after himself as he should of done”* (Roger, Aged 78*)*. Consequently, Roger was further determined to adhere to regular exercise having experienced this recent death. For Barbara (Aged, 67) the death of her husband signified a turning point, prompting her change what she described as *‘bad’* lifestyle choices. Barbara was unfortunate to lose her husband 10 years previously and then suffered her own cardiac complications; the combination of both events motivated her to be more physically active:

*I got the gypsies warning*. *I am sure fear played a lot in it*, *yes it did*. *My husband died when he was 63 and I mean he had a lot of illnesses*, *he finally died of a cardiac arrest…and that’s a dreadful shock*, *it takes you a long while to get over*, *erm*, *I really thought I was quite fit*, *it’s an on-going thing I think*

### Overcoming Aging

Overcoming ageing was identified as a significant theme. Sub-themes associated with overcoming aging were *independence*, *becoming a burden*, *functional fitness* and *isolation*.

#### Independence

**Fig 2:**
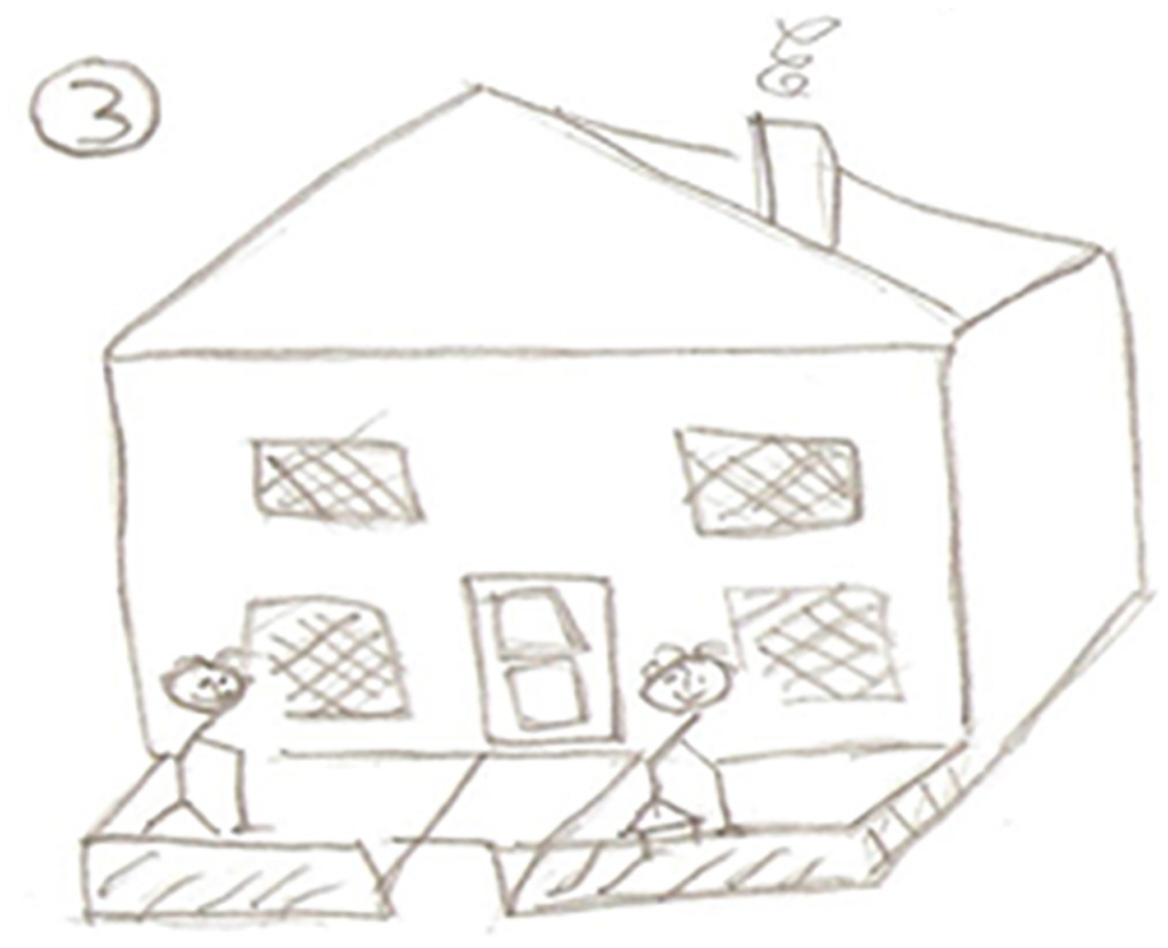
A picture of Independence.

Paul (Aged, 65) accentuated the need to exercise in order to remain independent: “*I know that if I keep fit that there is more chance that I will end up in my own home rather than going to a care home or a hospital or something else”* (See Fig 2). Mark was also motivated to remain independent: *“it’s a struggle if you’re not fit ok you can get one of those wheely things or get a wheelchair but I don’t want that”* (Mark, Aged 82).

Barbara also took a photograph of her home and cited independence as a motivator to maintain exercise: “*I want to retain my independence*, *I would dread ending up in a home*” (Barbara, Aged, 67).

#### Becoming a burden

Many participants did not want to become a burden on their families. Ian (Aged, 77) said: “*I want to make sure I am not a burden on them”* and Andrew (Aged, 83) said: “*I told my wife to shoot me if I have a stroke*...*it is not fair on her”*. For Barbara: “I would dread being a burden to my family and I realise if you want to enjoy, carry on as long as I can, I have got to keep fit” (Barbara, aged 67).

#### Functional fitness

Jason valued the exercise sessions in terms of how it helps to maintain his ability to move: *“a good reason for coming along here so you can keep fit and keep moving about*...*to keep yourself fit and mobile*”(Jason, Aged 71). Another participant states “*I’m now not too far off 90*, *I could run for the bus if I had to*, *I couldn’t run as fast as I used to but I could still run perhaps*’(Peter, Aged 87). The importance of such tasks has been described perfectly by Simon (Aged, 70) who describes them as, *“It’s the silly little things which to a young person*, *they wouldn’t think twice about*”. These participants appeared to value the benefits that exercise offers in order to maintain their independence and levels of functional fitness to carry out everyday tasks.

#### Isolation

Another sub theme of overcoming aging was isolation. Several participants commented that attending exercise classes reduced social isolation and promoted inclusion. Simon described aging as “*when you get older your life*, *the scope of your life contracts and there are fewer and fewer things you can do easily or activities that you can participate in and therefore small things become quite important*”. David also notes that

*there’s isolation out there for people of our age…we’re too young to go to an old people club and we are probably too young to go to a care home or something of that nature…there is a disconnection of one generation to the next…(and for) people of my age…for most of us if not all of us there is a degree of isolation which can be unpleasant*, *uncomfortable* (David, Aged 69).

Overcoming the physical decline of aging, the desire to remain independent and functionally fit in addition to avoiding social isolation were important motives for continued physical activity participation.

### Social Influences

Social influences were identified as a theme with sub-themes of being in the same boat, and, friendship and support. These sub-themes were directly related to the group nature of the exercise classes.

#### Being in the same boat

Participants were motivated to continue exercising to be amongst others ‘in the same boat’ who had experienced cardiac problems and felt a sense of relatedness to others. William finds common ground with his fellow exercisers, which spurs his motivation to take part: “*I feel more comfortable talking with people I know who are having the same problems and that so yeah that’s reassuring and it makes me want come*” (William, Aged 76). Mark also identified with other participants and enjoyed sharing experiences and listening to others’ stories: “*one of the greatest things is meeting other people of a like you know background*...*it’s great to converse with someone that’s had even worst situations than you or they have cured it or done something different and it’s just nice to know*” (Mark, Aged 82). Similarly, Harry emphasised:

*it’s very nice when you share it with somebody else*...*you can actually talk to other people about your experiences and listen to other people’s experiences*...*if you’re feeling a bit grotty that this happened and that happened and they say oh that has happened to me and I have now done this and it’s much better*, *it helps you to get over it easier I guess*. *It drags you through*...*just somebody else to talk to somebody else with the same experience helps (Harry*, *Aged 65)*

#### Friendship and Support

**Fig 3:**
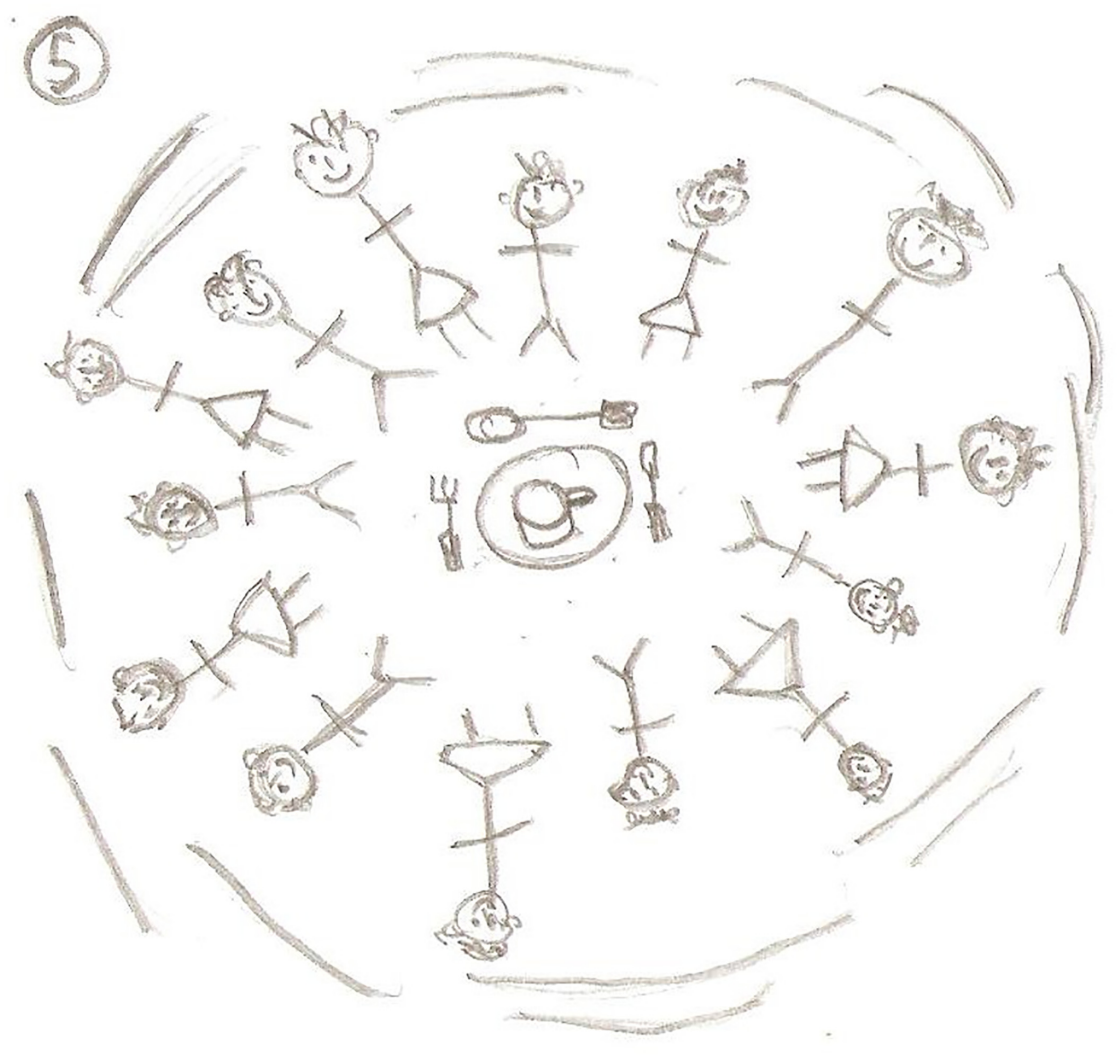
Exercise group socialise before the class.

Closely related to ‘being in the same boat’ was the sub-theme of friendship and support. Fig 3 depicts a sense of community, friendship and social gathering and represents the nature of this theme well; it also signifies the importance and reliance on others. Simon states:

*we socialise*, *not all the time but I think we depend on one another and we know that if we had a problem we could turn to one of them and they would help which is a great thing to know and if you’re not participating in any other major social group…this is probably their only social environment (Simon*, *Aged 70)*.

**Fig 4:**
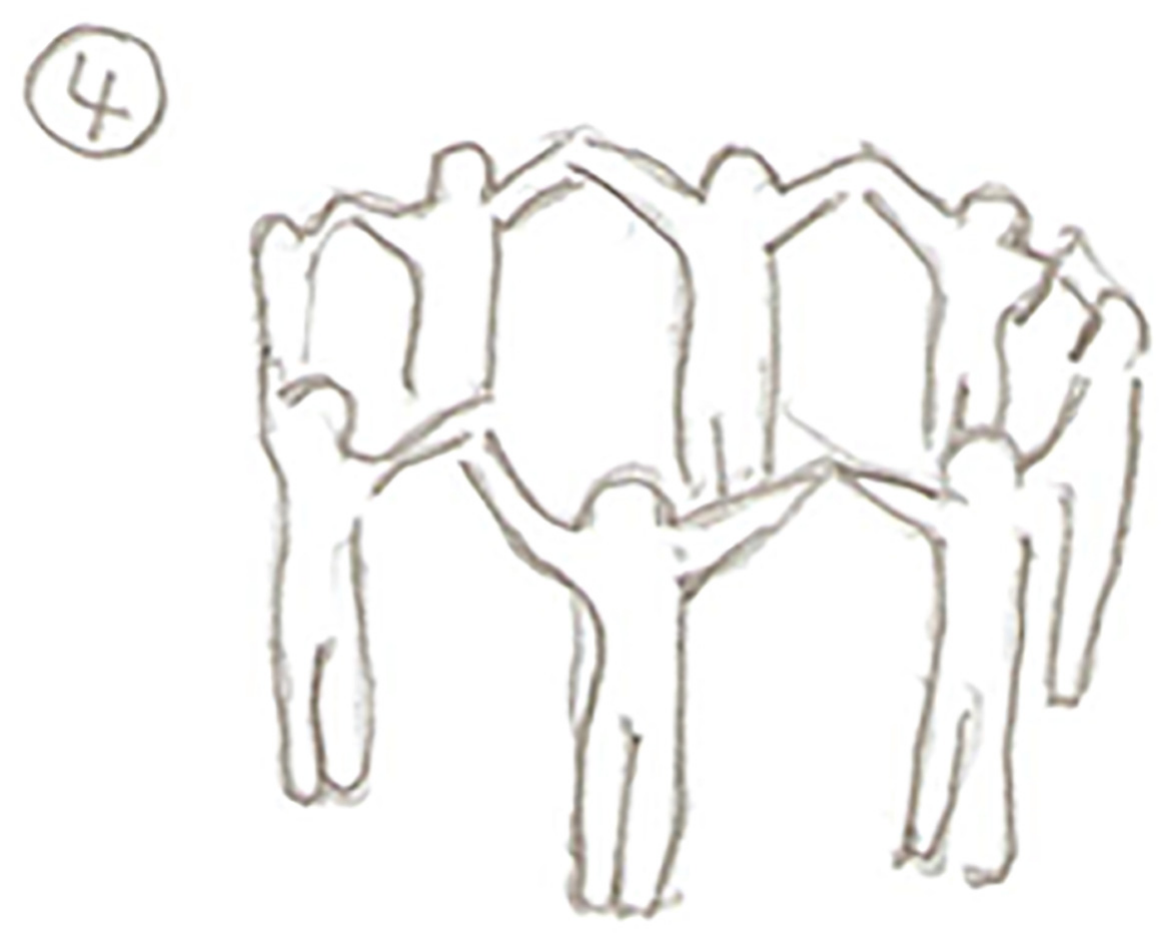
Carl’s drawing depicting friendship and support.

Friendship and socialising were regarded as exercise motives by many of the participants. These were often mentioned in succession of each other. Andrew happily declared “*the attraction is to coming down here to meet friends”* (Andrew, Aged 83). William emphasised *“I come here well for the company*...*so it’s very sociable*...*it’s the social aspect really”* (William, Aged 76). Carl similarly commented *“Well that is the socialising*. *You actually feel as though you’re among friends”* and “*it’s the social element as well*...*I think everybody needs to socialise*, *coming here every week you get plenty of it”* (Carl, Aged 69). Fig 4 is a picture Carl drew to represent the importance of being in a group and exercising together.

**Fig 5:**
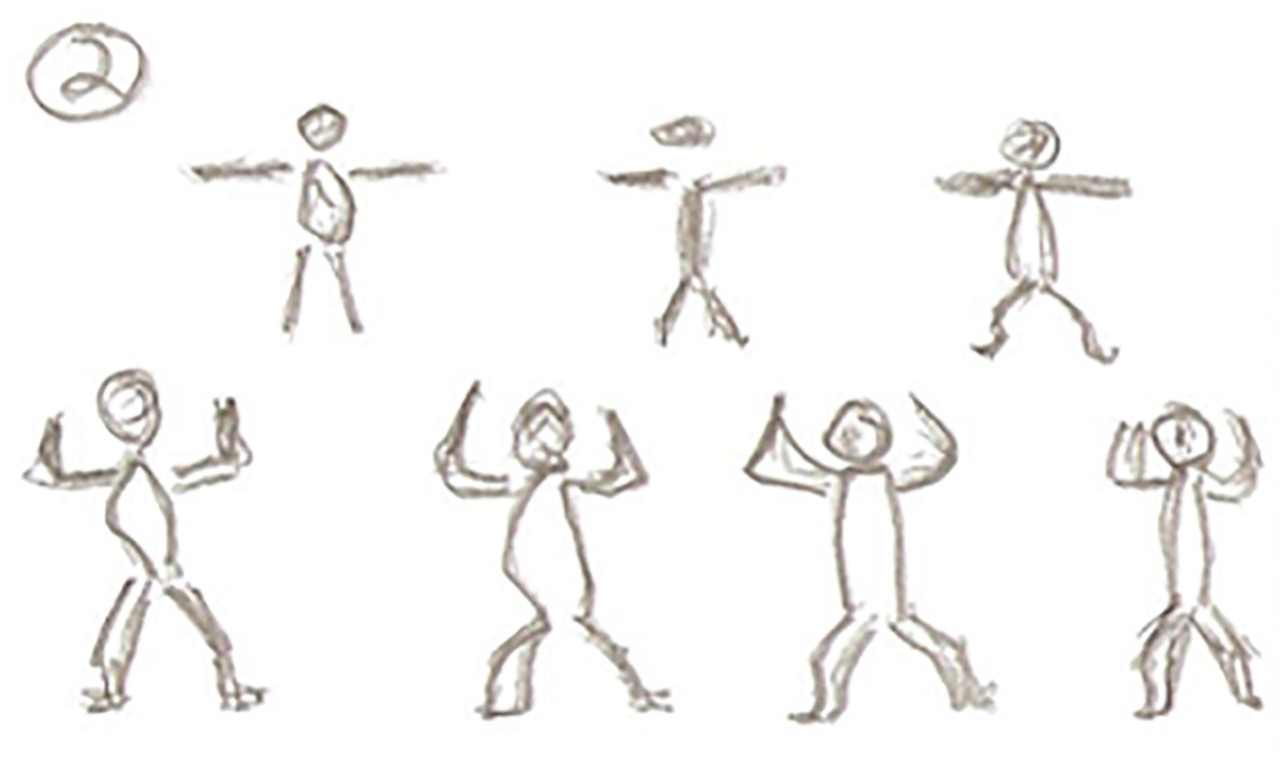
Exercising with others: a source of motivation.

Jim declared that the support of the class stimulates his attendance to the sessions. He commented *“so it is important that we socialise and support each other*...*then everybody’s doing it so you’re encouraging each other*”(Jim, Aged 63). Relative to support was exercising with others, Jason too was encouraged by the group: “*the fact that you’re doing it as a group you’re exercising as a group and that is an advantage”* and *“*...*the fact that you’re exercising with other people sort of gives you encouragement to do it more*, *it does actually motivate you to do it”* (Jason, Aged 71). Fig 5 is a drawing by Jason to reflect the importance of exercising with others.

## Being able to enjoy life

Another significant theme that emerged significantly in the photos and drawings was a sense of ‘being able to’. Sub-themes included being able to enjoy the good life and being able to spend time with family. Many participants referred to the need to be physically able to fulfil their personal pleasures.

### Being able to enjoy the good life

Being able to enjoy ‘the good life’ appeared to be a powerful motivator for several participants and was evident in the selected photographs. For Andrew, “*exercise is keeping me fit*, *and it’s keeping me fit that means I can go on an aer*oplane” (Andrew, Aged 83). For Andrew, being sufficiently fit and able to travel meant being able to have nice holidays: *“*being in the sun, having a nice time and being able to do it”. Paul and Mark both expressed their desire to be fit in order to fly across the world. Paul was motivated to exercise to *“*...*be able to travel and enjoy foreign holidays as well*...*to travel*, *to enjoy myself and do the things that I want to do with my wife”*. Two photographs were selected by Karen, with reference to the first Karen said: “*The reason to exercise is to keep fit to stay together and being able to do lovely things like this- walking in the Lake District*”. In reference to the second photo: “*This is in the West Indies so again it combines the exercise and being able to travel to go to nice places*”. In essence, being able to enjoy life into older age was a key motivator for wanting to remain physically active.

#### Being able to…spend time with family

Being able to spend time with family was another dimension of being able to and was portrayed in both the photographs and discussed further within the interviews. Several participants expressed the importance of their family as a motive for remaining physically active, whether it was to visit family abroad “*Well I need to be fit enough to travel and erm*, *go over there and see them*” (Karen, Aged 82) or to remain fit for their grandchildren; “*The idea of keeping fit is so you’ve got longer with your family*, *erm*, *grandchildren take a lot of exercise when they’re visiting so you have to keep up with them*” (Roger, Aged 78). And “*That’s my four grandchildren and that’s the stages their growing up…I want to see them grow up so that’s why I come to exercise to keep myself going so that I can see them grow up*. *I can do things with them as well you know*, *the fitter I am the more time I can spend doing things with them*” (William, Aged 76).

### Provision of routine and structure

The provision of routine, discipline and structure was particularly important and was achieved through regular attendance to the circuit classes. All participants previously completed structured cardiac rehabilitation programmes and through a continuation started to attend the circuit classes, in many cases this could be in excess of 5 years. For Matthew, the discipline and routine were deemed important: *“There’s some sort of discipline involved*, *if I was doing it myself erm*, *maybe I would say it’s raining I won’t go or something*, *whereas here I come when I possibly can*, *so it’s the discipline of it that helps”* (Mathew, Aged 71). Mark also referred to the set routine: “*it’s the discipline of doing it that makes it good because every Wednesday you think oh yes my day to exercise*” (Mark, Aged 82). Jason’s motivation derives from a feeling of accountability towards the group: “*When you know you have got to come here well I suppose it’s in a way because you think well other people are expecting me to come as well*” (Jason, Aged 71). A sense of duty and the structure offered by way of exercise classes appeared to increase commitment to and persistence in exercise for the participants.

### Enjoyment and Psychological Well-being

Enjoyment and psychological well-being were widely reported as motives by the participants for their maintained attendance. Paul consistently expressed the pleasure he obtained from attending the sessions. He conveyed that “*it’s enjoyable*...*it’s great fun to come here”* and *“*...*such good fun*...*I enjoy it so much”* (Paul, Aged 65). When referring to the sessions, Carl stated that *“I like it I enjoy it”* and *“I get so much enjoyment out of coming”* (Carl, Aged 69). Happiness also featured as a motive behind Harry’s participation. He conveyed *“Well that’s just happy*, *in a nut shell the sessions make me happy”* (Harry, Aged 65).

Although Andrew did not directly indicate that the sessions make him joyful, he still pointed out that if by attending the sessions he can achieve his goals i.e. weight loss, then he will be much happier. He phrased *“I will be much happier if I can achieve some or all of those of what I have previously said I want to aim for*...*you know instead of being potentially sad and miserable I would be happy and enjoyable”*.

**Fig 6:**
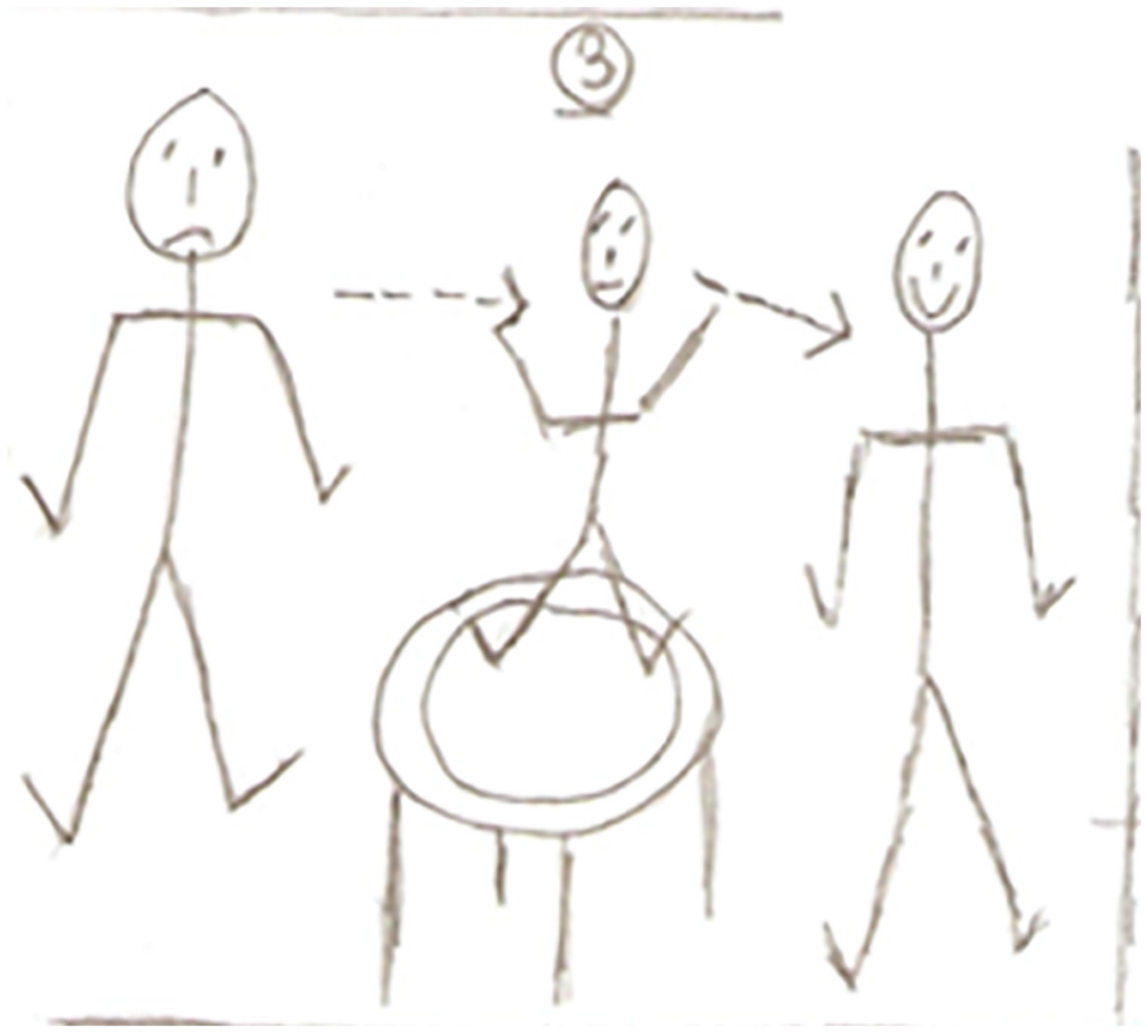
Bill’s improved mood following exercise.

Improved psychological well-being was also a motive and outcome of exercise. For Bill: *“Exercising makes me feel better*, *I love doing it*, *and I feel better afterwards…I feel more energetic actually afterwards*...*I really do feel good when I leave here*” (See Fig 6). Patrick similarly referred to improved well-being: *“it brings a bit of buoyancy… it really gives a boost”* (Patrick, Aged 59).

## Discussion

The present investigation aimed to explore the factors underlying maintenance of physical activity for participants that had completed cardiac rehabilitation at least two years previously. There were seven main themes identified as part of the analysis: *fear of death and ill health avoidance*, *critical incidents*, *overcoming aging*, *social influences*, *being able to enjoy life*, *provision of routine and structure*, *enjoyment and improved psychological well-being*.

The present study found that fear of death and illness avoidance were powerful motives for continued participation in exercise classes and participants felt that they had control over CVD risk factors and were able to make meaningful changes. The importance of perceived ability to exert control over the body and in relation to health has been reported elsewhere [1, 50–52]. Overcoming the physical decline of aging and a desire to remain independent and functionally fit were also important motives for continued physical activity participation. It would appear that fear and vulnerability alone is insufficient to motivate change but coupled with perceived control to take charge of health, participants were motivated to maintain physical activity and felt able to do so.

The second main finding was the impact of social influences on the maintenance of exercise. This is consistent with numerous studies that have found that the social nature of cardiac rehabilitation programmes facilitates continued participation [21–25]. In the present study, the sub-themes of social influences were being in the same boat, and, friendship and support. Participants were motivated to continue exercising to be alongside others who were perceived to be ‘in the same boat’ and had experienced similar cardiac problems. The importance of exercising with others’ who have had similar experiences has been reported elsewhere [21, 53–54]. The second sub-theme of social influences was *friendship and socialising* and these were regarded as key exercise motives by many of the participants. The importance of socialising found in the current study is significant since 78% of the sample were men. Although many studies claim that women are more likely to value the social connection and sense of relatedness gained through cardiac rehabilitation, there are some indications that men may equally enjoy this aspect [55]. The theme of social influences was directly related to the group nature of the exercise classes. Recent developments in cardiac rehabilitation have included more home-based physical activity programmes [56]. However, such home-based programmes are unlikely to foster relatedness to others or provide a social support network and these aspects appear to be powerful motives, outcomes and incentives for the longer-term maintenance of exercise amongst cardiac rehabilitation participants.

The third motive for maintaining participation was *being able to enjoy life*. These themes included being able to travel, spend time with family, and enjoy the good life and in many ways was linked to the desire to maintain a full and active life despite the process of aging. A similar finding was reported by Rogerson et al. [30] with several participants reported that ‘having a reason for exercising’ was an important facilitator for exercise maintenance. The ‘having a reason to exercise’ was associated with doing exercise for someone else other than themselves or exercising to improve health and live longer.

The provision of routine, discipline and structure was identified as a theme important for physical activity maintenance and this has been found elsewhere [57–59]. Martin and Woods [59] found that exercise classes were viewed as part of the weekly routine and provided cardiac participants with a sense of purpose. Hardcastle and Taylor [57] also found that planning exercise was a strategy that participants used for remaining committed to their new active lifestyle following participation in an exercise referral programme.

The final theme was *enjoyment and psychological well-being* and these were widely reported as motives by the participants for their maintained attendance. Rogerson et al. [30] also found that experiencing the psychological benefits of exercise was an important facilitator in maintaining physical activity. Other studies have also identified mental health benefits as a powerful motivator to maintain physical activity [57, 60]. In this way, experiencing mental health benefits from physical activity helps to make the exercise become an activity that is self-reinforcing.

The study used photo elicitation to explore exercise motives and could be used in future studies as an intervention tool. Several participant comments endorsed the added value in selecting pictures and the reflective process involved in being able to ‘show’ or articulate motives for continued exercise. We have included testimonies of the participants to illustrate how the photographs or drawings were able to facilitate more reflective thinking about motives for continued exercise. For example, “*I have hundreds of pictures I could show you…no one ever asked me to provide photographs of what exercise means to me before*, *it helped my identify how I feel about coming here*” (Matthew, Aged 71) and “*I think drawing them brought it home to me that it’s all about keeping myself fit*, *enjoying keeping myself fit so that I can enjoy the future*” (Paul, Aged 65). For another, the process of reflecting on motives made him realise his real driving motive for exercise: “*When I first thought about it the thing I thought that motivates me is the tea and biscuits…but in practice it is not that in itself what is really is the fact that I want to keep healthy*, *the tea and biscuits is the icing on the cake really it makes it a more attractive and enjoyable experience*” (Jason, Aged 71). As such, it may be worthwhile for future research to use photo-elicitation methods as an intervention tool to promote physical activity to those that are sedentary and not acting on their motives for change. Such methods have the potential to encourage more elaborated thinking about health behaviour and in the context of past, present and future.

### Limitations

Limitations of the current research should be acknowledged. Although our approach aimed to derive in-depth, rich data that explored the factors that influence motivation and commitment to continued exercise following participation in a cardiac rehabilitation programme, the sample was recruited from exercise classes in one location and the findings may not be transferable to other settings and participants. It should also be noted that participants were those that continued their exercise participation post cardiac rehabilitation. Group-based exercise may not foster adherence for everyone and further research should explore reasons for non-participation and discontinued participation in cardiac rehabilitation.

## Conclusion

The present study adopted a qualitative and visual methods approach to explore the factors that influence motivation and commitment to continued exercise following participation in a cardiac rehabilitation programme. A new finding was that ill-health avoidance was a powerful motive for exercise maintenance, but perhaps only when participants also value the outcomes of exercise and believe they are able to exert control over their health. Another novel finding that emerged from the pictures was the importance of *being able to* travel, spend time with family and go on holidays as a motive for continued exercise. The findings also have important implications for the design of future interventions. Interventions would do well to promote the outcomes of participation (improved health, independence, social inclusion, being able to enjoy life) and increase perceived control over health. The role of social influences supports the role of group-based exercise programmes in the cardiac population to promote relatedness, social inclusion and social support. Future interventions may be wise to use include peer role models to offer encouragement and to foster perceptions of competence in prospective participations. Interventions should also promote the social aspects of participation, and enjoyment to provide vicarious experiences to outsiders, that, in turn may nurture positive attitudes and confidence to exercise and future participation in cardiac rehabilitation programmes.
